# Examining the psychological effects of animosity on university students’ study choice intention

**DOI:** 10.1186/s40359-026-04552-z

**Published:** 2026-04-16

**Authors:** Dimin Wang, Yang Zhou, Juewei Lin

**Affiliations:** Faculty of Hospitality and Tourism Management, Macau University of Science and Technology, Avenida Wai Long, Taipa, Block O, Macao, SAR China

**Keywords:** Animosity, Country Image, International students, Higher education institution, Study choice intention

## Abstract

This study examines how Chinese undergraduate students’ feelings of animosity can be associated with their psychological perceptions and study choice intentions in the US. A total of 278 usable data responses were collected from Chinese students who enrolled in a private university in Macau, China; the data were analyzed through partial least squares-structural modeling (PLS-SEM) and necessary condition analysis (NCA) to create an expanded analytical process. PLS-SEM revealed that students’ overall animosity is positively associated with risk perceptions and negatively associated with their country image perceptions and study choice intentions toward the US. Moreover, NCA identifies various dimensions of animosity (i.e., economic, social, political, religious, historical, and military) as necessary conditions of students’ overall animosity. This study reveals that political and historical animosity are the two most important factors that are associated with students’ overall animosity. Actionable implications are offered to the US higher education institutions to understand students’ psychology and mobility in order to implement better strategies to recruit and retain Chinese international students.

**Supplementary Information:**

The online version contains supplementary material available at 10.1186/s40359-026-04552-z.

## Introduction

International education has long been appealing to Chinese students, enabling them to bypass grueling national exams and gain admission to top universities outside China [1, 2]. For the past 15 years or so, China was the top source market for international students in the US [3, 4]. However, more recently, the top status was surpassed by India, and the number of Chinese students has declined continuously over the years; the number dropped by more than 28% from *369*,*548* in 2019 to *265*,*919* in 2025 [5–7]. The sharp decline in student enrollment indicates that China’s favor for choosing the US as the study destination may be fading.

The decline in Chinese students’ enrollment in the US could be attributed to various reasons. First, it could be attributed to political reasons. Relations between China and the US are fraught with political disagreements and trade issues. Amid rising US protectionism, the US education sector has been politically weaponized against international students with non-Western backgrounds who are socially constructed to be unwelcome [3]. Moreover, the US government’s restrictive immigration policies have further obstructed international students’ entry into the US [8]. For instance, the US Congress initiated the “Stop CCP Visas” Act to stem the influx of Chinese international students whose universities are affiliated with the Chinese Communist Party, claiming students from these institutions pose a serious national security threat [9]. The implementation of strict student visa vetting procedures was further intensified after a Chinese engineer, Ji Chaoqun, who came to the US to study, was charged with espionage and jailed for eight years [10]. As such, thousands of student visas have been revoked [5, 6]. Another potential reason is that many Chinese students are concerned about their safety while studying in the US. For example, during the COVID-19 pandemic, many Chinese international students were scapegoated and discriminated for the origin of the coronavirus [4, 6]. Moreover, the Chinese public’s overall attitudes toward Western degrees have also shifted. For example, some Chinese employers, especially in civil service positions across public and government entities, may no longer favor applicants with foreign degrees. Reportedly, about 80% of Chinese students who graduated from US universities stated that their salaries fell short of their expectations, and 70% of them indicated that their offered positions were misaligned with their academic qualifications [11]. To exacerbate the current unfavorable situation, the Trump administration even imposed a hefty H1-B visa fee ($100,000) in September 2025 to deter foreign skilled workers from seeking employment in the US. This ludicrous restriction has further shrunk the talent pool of international graduates from entering US higher institutions [12]. In light of this, Chinese students’ choice to study abroad in the US has been greatly compromised.

Given the convoluted US-China relationship and rising nationalistic sentiment, this current study postulates that general animosity could inhibit Chinese students’ attitudes and behavioral intentions toward their study choice in the US. Animosity can arise from previous or ongoing military, political, and economic events [13]. Due to disparate political and economic systems, China and the US have constantly exchanged rhetoric and words of war. The Chinese media has consistently portrayed the US as an unfriendly nation seeking to contain China’s economic development [14]. As such, the general public in China who have been exposed to political propaganda news regarding the US-China relations tend to have negative perceptions toward the US, engendering high-risk perceptions for traveling to the US [15, 16]. Similarly, university students who harbored strong animosity toward certain countries would also be less likely to travel or study in those destinations if they deem them unfavorable [17]. Amid the geopolitical tensions, it is apparent that the soured US-China relationship could demotivate Chinese students’ intentions to study in the US [3]. Despite declines, some Chinese students’ motivation to study abroad may remain strong due to the reputation of US higher education institutions (HEIs) [18], and it is currently unknown to what extent animosity could override Chinese university students’ decision to study in the US.

Researchers in consumer and marketing research have revealed that animosity could determine consumers’ purchase intention if there is the presence of bilateral disputes among countries [19–21]. Later, this concept has been extended to the travel and tourism context, indicating that animosity could be associated with tourists’ travel intention and behaviors. Countries have been engaging in competition around the globe to attract tourists, investments, businesses, and talented residents, as well as competition for their higher education institutions [22]. International higher education is a multi-billion-dollar industry, which has been commercialized and treated as a commodity that is consumed rather than pursued. International students’ study abroad choices are strongly associated with commercial marketing approaches [23]. As such, one can argue that international students are “shopping around” for the best institution for their postgraduate studies. Therefore, we proposed that in the international higher education context, the degree or educational experience could be likened to a “product”, and the concept of animosity could be transferred and applied in the current study’s context to understand students’ study abroad choice intentions.

This study makes several contributions to the international higher education sector by shedding light on Chinese students’ perceptions of their study choice intentions in the US. Chinese students are invaluable to the US higher education, as they not only supply visible financial resources (nearly $40 billion) but also enrich the diversity of US college campuses [6, 23, 24]. This is especially true when diversity, equity, and inclusion programs (DEI) have been attacked under the Trump administration [25]. As such, it is critical for practitioners in higher education sectors to understand Chinese students’ psychology and perceptions to close gaps of misunderstanding and promote the US-China relationship under the current geopolitical environment.

Another novelty of this research lies in recognizing the application of the animosity concept in the higher education sector. In extant literature, while the discourse on animosity is well documented, it is mostly related to product consumption and international trade issues [26, 27]. However, in the international higher education context, students do not merely consume a service or product; as they enter a long-term commitment to a new socio-cultural environment, where their animosity perception could have negative long-term psychological effects and ramifications. However, the current discussion of animosity in the higher education context remains scarce [6]. We postulated that animosity under the international higher education context is usually directed at different targets, such as host institutions, the host country, and people in the host city. Given the nuance of animosity in higher education, it is critical to recognize its salient effects on international students’ psychological well-being. Applying the concept of animosity in the higher education context can offer new insight. Moreover, although previous research has categorized animosity in various dimensions, it is often fragmented [19, 20], and still limited research has encompassed holistic constructs to understand people’s animosity better. Finally, this study contributes methodologically by integrating partial least squares equation modeling (PLS-SEM) and necessity condition analysis (NCA) to enhance explanatory power and predictive accuracy, leading to a better understanding of students’ perception of animosity and their study choice intentions.

Based on the above information, this study addressed the following objectives: (a) propose a conceptual framework to explore inter-relationships among proposed variables; (b) examine the degree to which different animosity dimensions that can be associated with students’ overall animosity, and whether consequently overall animosity is negatively related to students’ country image evaluation, risk perception, and their study choice intention; (c) identify the necessary degree of determinants that are associated with students’ overall animosity, their evaluations toward the US, and their study choice intention.

## Literature review and research hypotheses

### Animosity

The concept of animosity has been applied across various disciplines (e.g., Marketing, Management, and Tourism) to understand consumers’ behavior in the context of bilateral disputes between countries [26, 28]. Animosity differs from ethnocentrism, as the latter describes the proclivity of individuals to view their own national group as the center of the universe [13]. People who hold ethnocentric views tend to denigrate a country’s products, claiming their own country’s products are superior despite their poor quality [13, 19]. However, people with animosity sentiments usually concur that products should be judged based on merit and quality, and they hold strong dislike and negative feelings toward a country, yet will still purchase products from that country [19].

Animosity describes individuals’ hostile attitudes towards specific parties, arising from previous or ongoing military conflicts or political events [19]. For example, animosity can be caused by warfare such as Japan’s invasion of China and Nazi Germany’s occupation during World War II [21]. Therefore, unlike adjacent constructs such as ethnocentrism, animosity is a unique construct that captures people’s negative perception toward a country due to deep-rooted emotions arising from past events [13]. Moreover, Jung et al. [20] further defined animosity as antagonism toward various groups arising from conflicts such as territorial, economic, and diplomatic disputes. For instance, South Korea harbored animosity toward the US and Japan during the Asian economic crisis in 1996 [13]. As such, animosity is standalone construct and could also be attributed to a particular situational event or stable incident over time involving long-lasting emotions [20].

However, consumers’ animosity toward a nation is predicated on a multitude of constructs from various causes [28]. Previous research has empirically and statistically validated that the overall animosity construct could be formed by different types of animosity [28, 29]. In addition to previously mentioned economic or military events, animosity can also be triggered by religious events, a country’s political system, and animosity towards a country’s citizens [28, 29]. Nevertheless, animosity has mainly been examined through the lens of tourism and consumer marketing research, and it warrants further investigation to determine whether this concept also applies to higher education settings.

In consumer research, feelings of animosity can overshadow consumers’ judgment of a product and decision-making [30]. Similarly, animosity in Chinese students’ psychological processes will likely play a role in shaping their antagonism toward the country where they plan to study overseas. Li et al. [6] examined Chinese students’ attitude towards US higher educational institutions through the lens of animosity; however, that study examined animosity only through the economic lens and students’ general antagonistic feelings. Building on previous research, this study adopts a more holistic scale of animosity (i.e., economic, social, political, religious, historic, and military) to capture Chinese students’ overall animosity toward the US [28, 29]. We postulate that different types of animosity could be associated with students’ perception of overall animosity. Accordingly, the following hypotheses were formulated:

- H1-H6: 1) Economic 2) Social 3) Political 4) Religious 5) Historical 6) Military animosity could be positively associated with overall animosity.

### Country image

Country image is an aggregate of different attributes, such as attractions, infrastructure, political image, economic image, and environmental image [31, 32]. In consumer research, the country image represents a brand image for consumers to purchase products made from specific countries [33]. Srikatanyoo and Gnoth [34] defined country image as a country’s industrialization, national standards, and other information related to its services and products. In the higher education field, a country’s image reflects its culture and social environment, which subsequently impacts the reputation of its higher educational institutions [6, 35]. As such, country images are among the critical motivating factors influencing international students’ study abroad intention [31].

### Overall animosity and country image

The extant research has shown that animosity is negatively related to people’s judgment [26]. For example, Souiden et al. [32] found that Chinese consumers’ animosity could be negatively related to the perceived product quality of Taiwanese brands and their willingness to purchase. Moreover, Latif et al. [35] found that Chinese consumers’ animosity toward the US is closely related to their reluctance to purchase US products. Several studies in tourism also indicated that animosity could be negatively associated with tourists’ evaluation of country image and destinations [28, 36, 37]. Thus, it can be postulated that students’ perceptions of animosity could be negatively related to the host country’s image. Accordingly, we proposed:

- H7: Chinese students’ overall animosity could be negatively associated with the US

### Animosity and study choice intention

Recent studies have found that animosity could be negatively associated with people’s visit intentions [38]. For example, Choe [30] revealed that the Korean people’s animosity hindered their visits to Japan. Similarly, Chinese students’ animosity sentiment could be potentially related to their attitudes and behavioral intentions toward studying in the US. For instance, Li et al. [6] found that Chinese students’ animosity could have a negative correlation with their attitudes toward US higher institutions. Moreover, drawing on intergroup research [39, 40], when there is the presence of animosity, Chinese students tend to anticipate divergence in their identity from the common ingroup (the US university environment), and consequently reduce their sense of belonging to the host community. As such, animosity could intensify the feelings of in-group identity [41], and students influenced by in-group identity may consider studying in countries culturally close to them. Accordingly, we proposed:

- H8: Chinese students’ overall animosity could be negatively associated with their study choice intention in the US.

### Animosity and risk perception

The strained foreign relationship can be a risk factor for people to visit abroad [42]. Risk perception is generally defined as the likelihood of dangerous events that are beyond one’s ability to control [43]. Previous studies have found that animosity can negatively affect people’s risk perceptions. For instance, Alvarez et al. [15] revealed that when people’s animosity increases, their risk perception also increases. Furthermore, Abraham and Poria [38] found that animosity could be positively associated with people’s perceived risk. Although the linkage between animosity and risk perception has not been substantiated in the higher education context, it could be inferred that Chinese students’ overall animosity toward US policies or rhetoric could translate into negative intergroup affect (e.g., fear of discrimination, anxiety about safety) [40]. Accordingly, we proposed:

- H9: Chinese students’ overall animosity toward the US positively affects risk perception.

### Country image and study choice intention

The country image could be associated with students’ intention to study abroad. For instance, Nghiêm-Phú and Nguyễn [31] found that Vietnamese students are more likely to study abroad if they hold a positive image of the host country. Western countries such as the US can be appealing to international students seeking to pursue graduate education due to employment opportunities after graduation [6]. However, Chinese international students may have mixed emotions toward studying in the US. On the one hand, Chinese youth have enjoyed US entertainment and admired its technological innovation and educational opportunities, while on the other hand, US policy has impeded Chinese students from forming favorable perceptions of the US [6]. The bilateral relationship between the host country and China can dictate students’ choices of study destination [44]. Country images have long been considered paradoxical [45]. As such, Chinese international students may perceive the US positively as a destination with plentiful educational opportunities, while simultaneously perceiving the country negatively at the macro and political level. Given the political rhetoric, it is currently unclear whether the US country image exerts any positive effects on Chinese international students’ study choice intention. Thus, it is proposed:

- H10: Country image of the US could be positively associated with Chinese students’ intention to study in the US.

### Research model

**Fig. 1:**
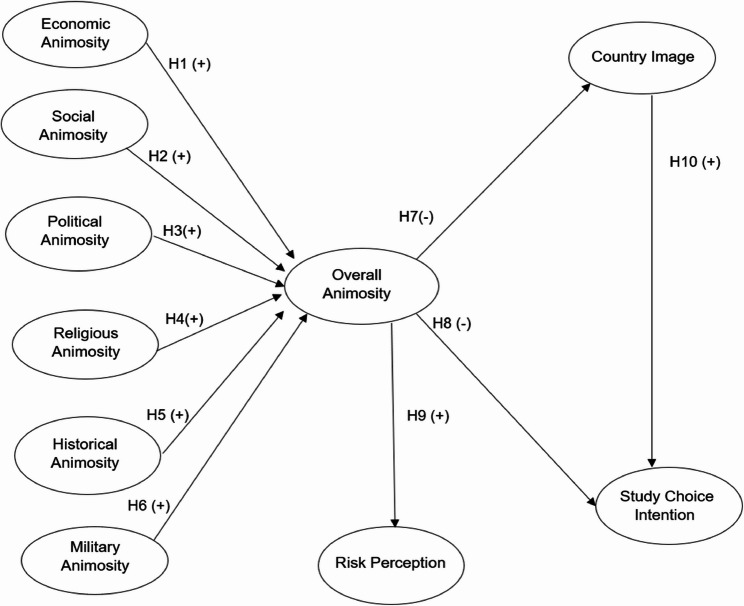
Study model

The research model (Fig. 1) derives from the hypotheses development, consisting of 10 constructs. The directionality for H1-H6 is positive, whereas the directionality for H7 and H9 is negative. The directionality for H10 is positive.

## Research methods

### Data collection

Given the time and location constraints, this study employed a non-probability sampling strategy (i.e., convenience sampling) to recruit students to participate in the survey. Online surveys were employed to carry out data collection via Google Forms, and the survey links were mainly sent to tourism major students who enrolled in a few similar courses from a private university in Macau, SAR, China. Since the sample surveyed in this study is context-specific rather than representative of the broader population of Chinese university students, the generalizability of this current study is limited. The questionnaire was initially prepared in English and subsequently translated into Chinese to facilitate data collection. The translation followed a back-to-back translation process to ensure the validity of questionnaire items [46]. Participants were offered extra course credit to encourage participation and obtain an adequate number of responses. Participation was voluntary, and respondents could exit the survey at any time without submitting their responses. A 25-pilot test was carried out to ensure the content validity of the questionnaire survey and identify any technical issues. The official full-scale data collection was launched between October 9th and November 22nd in 2025. A total of 284 responses were collected; however, 278 usable responses were retained for final analysis after cleaning the six problematic data points (e.g., straight-liners and surveys completed prematurely).

### Measurement items

The measurement items (Appendix 1) were adapted from prior research. Animosity constructs contain 17 items, which were adapted from Campo and Alvarez [28]. and Sanchez et al. [29]. Among the animosity constructs, economic, social, political, and military animosity contain three items, respectively; historical, religious, and overall animosity contain two items, respectively. Four items of country image were adapted from De Nisco et al. [26]. Risk perception contains two items that were adapted from Tavitiyaman and Qu [47]. The study choice intention was adapted from Guo [2], To et al. [48], and Zhuang et al. [49], containing three items in total. The measurement scales were anchored on a seven-point Likert scale (1 = strongly disagree; 7 = strongly agree). The last part of the questionnaire asked for respondents’ demographic information.

### Data analysis procedures

SmartPLS 4 software was used to carry out both PLS-SEM and NCA analysis [50]. PLS-SEM is a regression-based procedure, focusing on maximizing the variance of the unexplained construct [51, 52], which follows the sufficiency logic to explain whether “a higher X leads to a higher Y” [53]. Compared to covariance-based SEM, PLS-SEM is an efficient method to identify important explanators in models and maximize prediction, since PLS-SEM reduces residuals of dependent constructs in the model [51, 54]. Triangulating PLS-SEM with NCA is an emerging approach [54]. NCA follows necessity logic, indicating the effect will not occur without achieving a certain level of causal factor (“X is needed for Y”) [52, 53]. The prediction results will be incomplete if necessary conditions are not considered [55]; hence, PLS-SEM and NCA are complementary to each other. G*Power 3.1.9.7 software was used to calculate the minimal sample size for the PLS-SEM analysis [56], which 160 samples were recommended by the software as the minimum sample size. Moreover, according to Dul [55], NCA in quantitative research typically requires a sample size of 180 to have sufficient power to detect necessary conditions. Thus, the samples collected in this study are adequate for analysis.

### Common method bias

To mitigate common method bias (CMB), survey items were placed in different orders during the data collection and were adopted from various sources [57]. Moreover, Harman’s single-factor approach via principal component analysis was carried out to assess the CMB issue [58]. The result showed 32.96% variance accounted for by the first factor, which was lower than the cut-off value of 50%. Moreover, a full collinearity test was conducted, in which VIF values for all latent variables were lower than 3.3, indicating this research was not contaminated by CMB [59]. Based on the above arguments, the CMB issue was minimized in this study.

## Findings

### Respondent characteristics

Table 1 describes respondents’ general information. In terms of gender, males account for 38.8% (*n* = 108), while females account for 61.2% (*n* = 170). Since all participants are undergraduate students, their ages are 18–23 years old. The majority of the students major in hospitality and tourism management (65.1%, *n* = 181). Also, more than 95% of respondents were from the Chinese mainland. Excluding US as a potential study destination, many students indicated that they preferred to further their education in Hong Kong (37.1%, *n* = 103), followed by UK (24.5%, *n* = 68), Australia (11.9%, *n* = 33), Singapore (10.1%, *n* = 28), as well as other European countries (6.8%, *n* = 19).

**Table 1:** Respondents’ characteristics

Description	Frequency (no.)	Percentage (%)
Gender		
Male	108	38.8
Female	170	61.2
Total	278	100
Age		
18–20	186	66.9
21–23	92	33.1
Total	278	100
Major		
Hospitality and Tourism Management	181	65.1
Business and Finance	48	17.3
Science and Engineering	5	1.8
Humanities and Arts	34	12.2
Medical Science	5	1.8
Law	5	1.8
Total	278	100
Hometown		
Macau	47	16.9
Taiwan	2	0.7
Mainland China	229	82.4
Total	278	100
Study Destination Preference (non-US)		
Hong Kong	103	37.1
Australia	33	11.9
UK	68	24.5
Canada	5	1.8
New Zealand	4	1.4
Singapore	28	10.1
Southeast Asia (Malaysia, Thailand, and the Philippines)	1	0.4
European Continent	19	6.8
Other	17	6.1
Total	278	100

### PLS-SEM measurement model

Reliability and validity were checked to assess the measurement model (Table 2). Almost all indicator loadings were above 0.7 (one indicator happened to be 0.7); all average variance extracted (AVE) were above 0.5, implying the establishment of convergent validity. The values of Cronbach’s alpha were all above 0.7, and all composite reliability (CR) were above 0.7, demonstrating good internal consistency [51]. The discriminant validity was assessed by using Fornell and Larcker’s criteria to confirm whether the square root of AVE for each construct is greater than any correlations among the variables [60]. Table 3 shows the mean, standard deviation, and indicator loadings for measurement scales.

**Table 2:** Reliability and validity

	α	CR	AVE	CI	EA	HA	MA	OA	RP	PA	RA	SA	SCI
CI	0.72	0.82	0.53	0.73									
EA	0.87	0.92	0.79	0.12	***0.89***								
HA	0.89	0.95	0.90	-0.01	0.41	***0.95***							
MA	0.82	0.89	0.73	0.11	0.51	0.58	***0.85***						
OA	0.93	0.97	0.94	-0.27	0.31	0.63	0.43	***0.97***					
RP	0.76	0.89	0.80	-0.15	0.29	0.42	0.34	0.46	***0.90***				
PA	0.83	0.90	0.75	-0.17	0.42	0.61	0.44	0.65	***0.43***	***0.86***			
RA	0.77	0.90	0.81	-0.10	0.29	0.51	0.40	0.48	0.41	0.56	***0.90***		
SA	0.72	0.84	0.64	-0.10	0.50	0.59	0.46	0.59	0.35	0.67	0.48	***0.80***	
SCI	0.91	0.94	0.85	0.30	-0.10	-0.34	-0.15	-0.43	-0.24	-0.21	-0.15	*-0.35*	***0.92***

The italic value displays the square root of AVE value

**Table 3:** Mean, standard deviation, and indicator loading of measurement items

Items	M	SD	Loadings
Economic Animosity			
EA1	5.22	1.34	0.91
EA2	4.82	1.43	0.91
EA3	5.41	1.28	0.84
Social Animosity			
SA1	3.80	1.28	0.80
SA2	4.41	1.45	0.82
SA3	4.73	1.58	0.79
Political Animosity			
PA1	4.31	1.28	0.87
PA2	4.10	1.41	0.91
PA3	3.70	1.47	0.80
Religious Animosity			
RA1	3.50	1.35	0.91
RA2	3.82	1.35	0.89
Historic Animosity			
HA1	4.10	1.49	0.95
HA2	4.20	1.50	0.95
Military Animosity			
MA1	4.91	1.36	0.88
MA2	5.40	1.41	0.80
MA3	5.41	1.39	0.88
Overall Animosity			
OA1	3.69	1.41	0.97
OA2	3.36	1.47	0.97
Country Image			
CI1	5.85	1.14	0.70
CI2	5.54	1.26	0.75
CI3	5.71	1.11	0.73
CI4	4.64	1.28	0.74
Risk Perception			
RP1	4.26	1.29	0.90
RP2	4.32	1.31	0.89
Study Choice Intention			
SCI1	4.50	1.66	0.90
SCI2	3.70	1.73	0.93
SCI3	3.62	1.78	0.93

### Hypotheses testing-PLS-SEM

**Fig. 2:**
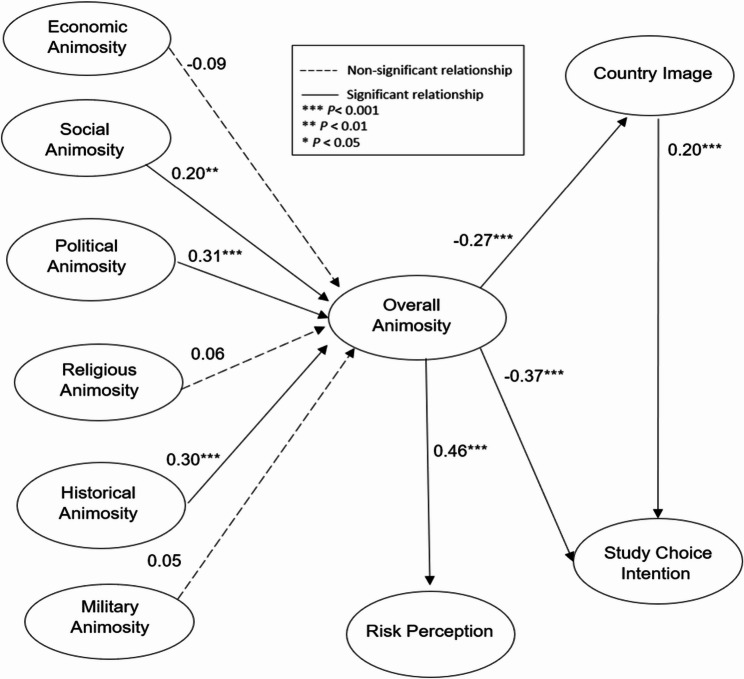
Results of PLS-SEM analysis

Bootstrapping with 5,000 samples was used to assess the significance of the proposed hypotheses [53]. The t-statistics (t > 1.96) was used to test the hypotheses [52]. Seven out of ten hypotheses were supported. Table 4; Fig. 2 display the results of the PLS-SEM analysis.

**Table 4:** The results of the hypothesized model

	β		t-value	Results
H1 Economic Animosity→ Overall Animosity		-0.09	1.45	Not Supported
H2 Social Animosity → Overall Animosity		0.20**	2.97	Supported
H3 Political Animosity→ Overall Animosity		0.31***	4.38	Supported
H4 Religious Animosity → Overall Animosity		0.06	1.01	Not Supported
H5 Historical Animosity→ Overall Animosity		0.30***	4.47	Supported
H6 Military Animosity → Overall Animosity		0.05	0.69	Not Supported
H7 Overall Animosity→ Country Image		-0.27***	5.04	Supported
H8 Overall Animosity→ Study Choice Intention		-0.37***	6.27	Supported
H9 Overall Animosity → Risk Perception		0.46***	7.78	Supported
H10 Country Image → Study Choice Intention		0.20***	3.58	Supported

Note.**p < 0.01, *** *p* < 0.001

### Results of NCA analysis

**Fig. 3:**
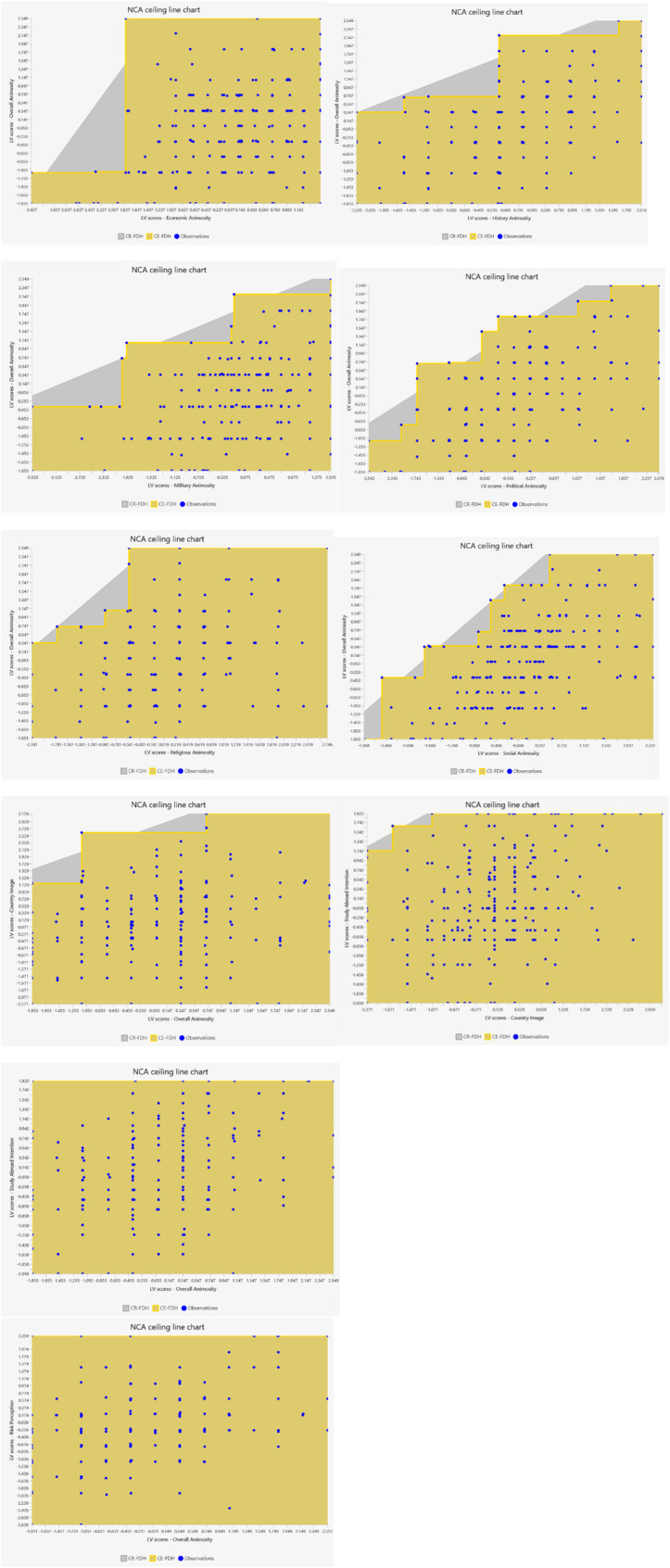
NCA scatter plots

The latent scores from PLS-SEM and 10,000 permutations were employed for NCA analysis [53]. Three iterations were carried out to test the necessity conditions. Conditions with the effect size less than 0.1 or p-value greater than 0.05 indicate that there are presence of not necessary conditions [55]. (Table 5). Moreover, NCA uses the ceiling regression-free disposal hull (CR-FDH) line to determine the minimum level of X needed to reach a certain level of Y [53, 61]. Figure 3 shows NCA scatter plots.

**Table 5:** NCA effect sizes

Construct	Overall Animosity Effect size (d)	*p*-value	Country Image Effect size (d)	*p*-value	Risk Perception Effect size (d)	*p*-value	Study Choice Intention Effect size (d)	*p*-value
EA	0.27	0.01						
SA	0.31	< 0.001						
PA	0.30	< 0.001						
RA	0.14	0.001						
HA	0.26	< 0.001						
MA	0.35	< 0.001						
OA			0.10	0.18	< 0.001	0.55	0.02	0.10
CI							< 0.001	< 0.001

Meaningful necessary conditions: d ≥ 0.1; *p* < 0.05

The bottleneck table (Table 6) is used to present the ceiling line result of the necessity condition threshold to reach outcomes [53]. As indicated by the bottleneck table, it requires 8.63% of economic animosity, 71.22% of social animosity, 88.49% of political animosity, 92.09% of historic animosity, 88.49% of military animosity, and 33.45% of religious animosity to achieve 100% overall animosity, respectively. The interpretation of comparative results of PLS-SEM and NCA analysis is shown in Table 7.

**Table 6:** Bottleneck table (percentage-levels)

Overall Animosity							Country Image	Risk Perception	Study Choice Intention	
Y (%)	Economic Animosity	Social Animosity	Political Animosity	Historic Animosity	Military Animosity	Religious Animosity	Overall Animosity	Overall Animosity	Country Image	Overall Animosity
0.00	0	0	0	0	0	0	0	0	0	0
10.00	0	0	0	0	0	0	0	0	0	0
20.00	0.72	1.08	0	0	0	0	0	0	0	0
30.00	0.72	2.16	1.44	0	0	0	0	0	0	0
40.00	1.08	3.96	5.04	0	1.44	0	0	0	0	0
50.00	1.44	6.84	8.63	4.32	1.80	5.76	0	0	0	0
60.00	2.52	11.51	19.07	10.79	4.32	6.84	0	0	0	0
70.00	2.88	19.42	28.42	22.66	9.35	12.95	0	0	0	0
80.00	4.77	30.22	62.23	60.79	31.66	16.19	20.86	0	0	0
90.00	6.48	55.76	81.29	80.58	65.83	19.78	42.45	0	5.04	0
100.00	8.63	71.22	88.49	92.09	88.49	33.45	76.26	0	11.87	0

**Table 7:** Interpretation of comparative result of PLS-SEM and NCA

Scenario	PLS-SEM results	NCA results	Conclusion
EA → OA	Nonsignificant determinant	Necessary condition	A level of 8.63% of economic animosity is necessary for overall animosity to manifest; however, a further increase will not lead to an increase in overall animosity.
SA→ OA	Significant determinant	Necessary condition	An increase in social animosity will increase overall animosity; however, a level 71.22% of the social animosity is necessary for overall animosity to manifest.
PA→ OA	Significant determinant	Necessary condition	An increase in political animosity will increase overall animosity; however, a level 88.49% of the political animosity is necessary for overall animosity to manifest.
RA → OA	Nonsignificant determinant	Necessary condition	A level 33.45% of the religious animosity is necessary for overall animosity to manifest; however, a further increase will not lead to an increase in overall animosity.
HA→ OA	Significant determinant	Necessary condition	An increase in historic animosity will increase overall animosity; however, a level 92.09% of the historic animosity is necessary for overall animosity to manifest.
MA→ OA	Nonsignificant determinant	Necessary condition	A level of 88.49% of the military animosity is necessary for overall animosity to manifest; however, a further increase will not lead to an increase in overall animosity.
OA → CI	Significant determinant	No necessary condition	An increase in overall animosity will negatively increase perception of country image; no minimum level of overall animosity is necessary to lead to negative country image perception.
OA → SCI	Significant determinant	No necessary condition	An increase in overall animosity will negatively increase in intention to study in the US; no minimum level of overall animosity is necessary to lead to negative intention to study in the US.
OA→ RP	Significant determinant	No necessary condition	An increase in overall animosity will negatively increase risk perception toward the US; no minimum level of overall animosity is necessary to lead to risk perception.
CI→ SCI	Significant determinant	No necessary condition	An increase in country image can increase intentions to study in the US; no minimum level of country image is necessary to lead to intention to study in the US.

## Discussion

This study examined whether different aspects of animosity could form Chinese students’ overall animosity and subsequently affect their image and risk perception as well as study choice intentions toward the US. The proposed research objectives have all been achieved, and the study has confirmed that students’ overall animosity is negatively associated with students’ country image and risk perceptions, as well as study choice intentions toward the US. Moreover, the dual analysis of PLS-SEM and NCA offered a unique perspective of the different dimensions of animosity that could contribute to students’ overall animosity, providing actionable practical implications for Higher education institutions (HEIs) in the US.

Based on the PLS-SEM analysis, the study found that social and political animosity positively contributed to overall animosity, demonstrating that one’s perception toward a country’s international politics and the hostility of its people can increase one’s overall animosity [62]. Moreover, political animosity has the strongest effect (*β* = 0.31) on overall animosity, validating that political policies and the political system of a country constitute important causes of one’s overall animosity [20]. Moreover, historical animosity also has a strong effect (*β* = 0.30) on overall animosity, revealing animosity can be triggered by past historical events and international conflicts [28]. The US is often accused by the Chinese authorities of meddling with China’s internal affairs (e.g., the prolonged Taiwan issue) [63]; thus, Chinese people may feel antagonistic toward the US in general. Surprisingly, economic animosity did not positively increase overall animosity. A possible explanation is that China remains the US’s largest trading partner and is the beneficiary of US investment and management expertise since China’s open-door policy in the late 1970s, which greatly boosted China’s economic development [6]. As such, despite being a major adversary of the US, China has also reaped great economic benefits from the US. Moreover, the draconian tariff policy initiated by the Trump administration caused only minimal impacts, as China heaped $111.68 billion trade surplus as of November 2025 [64]. Coupled with these reasons, we could expound the reason why economic animosity exerted a minimum effect on people’s overall animosity.

Furthermore, the PLS-SEM findings revealed that religious and military animosity did not significantly increase students’ overall animosity. This could be understood as China is an atheist society; hence, Chinese students in general are nonchalant towards religious affairs in the US. Regarding military animosity, unlike China’s convoluted relationship with Japan, China and the US have never engaged in direct military confrontation; as such, Chinese students may not have direct negative feelings toward the US military’s actions in the world. Moreover, even though the current US involvement in the Middle Eastern region has received criticism and condemnation internationally, Asian students in general may not form a deep military animosity toward the US in general. This may be due to country distance [22], as the Confucian East Asia is culturally and geographically distant from Middle Eastern countries.

Additionally, aligning with previous studies [15, 28, 30], the study has suggested that overall animosity could be negatively associated with image perceptions and behavioral intentions, demonstrating Chinese students’ general sentiment of animosity toward the US. As a corollary, this study corroborated that animosity could feed people’s risk perceptions [15, 37], revealed a positive relationship between animosity and risk perception,

Based on the NCA results, the proposed dimensions of animosity (economic, social, political, religious, historical, and military) are necessary conditions of students’ overall animosity toward the US. This result further corroborates that the nature of animosity is complex and contains various types with different dimensionality [29]. Although economic animosity, religious animosity, and military animosity are not direct determinants of people’s overall animosity based on average-based analysis (i.e., PLS-SEM), necessary conditions in degree are required to manifest the outcome (i.e., economic animosity, religious animosity, and military animosity ). Additionally, PLS-SEM revealed that an increase in overall animosity will have a positive association with people’s negative perception toward the country image, despite overall animosity not being a necessary condition in degree for country image based on NCA. This highlights the importance of decreasing people’s overall animosity to enhance their image perceptions of a country.

Furthermore, overall animosity is not a necessary condition of students’ intention to study in the US; however, it is a significant negative determinant of their intentions based on PLS-SEM. As such, it demonstrates that Chinese students’ general animosity hampers their intention to study in the US, as an increase of any degree of animosity (no minimum level) would negatively affect their intention to study in the US. Moreover, overall animosity is not a necessary condition of risk perception; however, it is a significant determinant of risk perception based on PLS-SEM analysis. As such, it is important to lower animosity, as a slight degree increase in overall animosity may spike people’s risk perception. Lastly, an increase in country image can increase intention to study in the US; however, no minimum level of country image is required to achieve the outcome. This is understandable, as the US universities can provide good educational resources with professional prospects and development in the future [3, 6]. Therefore, Chinese students’ intentions to study in the US are generally motivated by extrinsic factors such as studying at top universities and gaining good educational resources to bypass grueling college entrance exams [33], corroborating the paradoxical nature of country image [44]. As such, Chinese students’ perception toward the US remains largely positive.

### Theoretical implication

This study provides several theoretical contributions. Firstly, this study advances the extant literature on animosity in the international education context, while most of the previous studies are conducted in other social science disciplines, such as consumer marketing [28, 63]. As such, this study provides evidence that animosity can be applied in international higher education to capture its holistic dimensions. Little research has been conducted in a similar manner to incorporate different aspects of animosity to examine Chinese students’ perceptions and study-choice intentions toward the US [6]. The conceptualization of animosity could benefit from a typology that identifies various forms of hostility [65]. This study verifies that the concept of animosity is multi-dimensional, encompassing various types, providing future studies with solid evidence that causes of animosity need to be expanded further [21, 29].

Furthermore, the integration of the joint PLS-SEM and NCA approaches provides a better understanding of whether the presence of necessary conditions can predict the desired outcomes [53, 66]. For instance, PLS-SEM revealed that social, political, and historical aspects of animosity affected students’ overall animosity; however, these constructs need to reach a certain level to exert efficacy to impact the outcomes. Moreover, NCA was able to identify that all animosity elements are necessary conditions that contribute to students’ overall animosity, despite some of them being non-significant determinants (i.e., economic, religious, and military animosity) based on PLS-SEM output. However, these non-significant but necessary conditions can still manifest the outcome if they reach certain levels of degree. As such, NCA can help researchers to “gain a better understanding of causal effects when testing a particular outcome” [55, p2]. The combination of PLS-SEM and NCA demonstrates that not only significant but also non-significant determinants can constitute necessary conditions [53], offering new insights into causal mechanisms to test alternative theoretical arguments [54].

### Practical implications

This study provides several practical implications for US higher education institutions (HEIs) seeking to recruit and support international students. Given that overall animosity was negatively associated with students’ intentions to study in the US, the US HEIs should interpret this finding cautiously and focus on institution-level responses that are within their control. Rather than attempting to alter broad country-level attitudes directly and completely, universities may benefit from strengthening institutional trust, communicating more clearly with prospective students, and creating supportive academic and social environments.

While it is impossible to eliminate students’ antagonistic views, US higher education institutions may reduce the levels of overall animosity to a degree that engenders minimal negative effects to alter students’ perceptions. For example, the US HEIs can facilitate informal cultural exchange, provide clear information about student support services, highlighting a welcoming learning environment for Chinese and other international students. It is important for the US HEIs to address students’ animosity sentiment. As noted by Borinca et al. [40], experiences of exclusion push minorities to seek belonging within their own group. Therefore, animosity could potentially trigger international students’ reinforcement of ingroup identity, serving as the impetus for Chinese students to choose to study domestically or anywhere that is culturally proximate.

By addressing social animosity, students’ overall animosity could be more easily contained. As such, the US HEIs may enhance prospective students’ confidence by promoting an inclusive campus climate, demonstrating respect for cultural and religious diversity, and providing visible support mechanisms that help international students adapt academically and socially. These efforts may not directly reduce broad political or historical animosity toward the US, but they may help strengthen students’ trust in the institution itself. Although DEI programs in certain universities in the US have been disbanded, US HEIs in general can still facilitate informal cultural exchanges among international students and can promote positive images and showcase the friendly learning and social environment for Chinese students to study in the US.

Regarding the fact that political animosity and historical animosity are both significant determinants and necessary conditions, the US HEIs need to find a way to counteract international students’ exposure to negative messages in relation to US political issues and historical events to reduce the negative impressions of the US. This is consequential, as they affect students’ learning experiences in the US. Nevertheless, it is also relatively easy to implement measures to curb these two dimensions of animosity, as they both require high levels to manifest overall animosity. As long as these two animosity factors’ levels of animosity are contained in certain degrees, students may not form their overall animosity. As such, in practical terms, it is recommended for US HEIs to highlight national strengths and the strong position of studying in the US [67] and downplay negative news regarding the US government’s policies or involvement in different regions globally, as well as how geopolitical tension affects international student mobility.

Regarding economic animosity, the US HEIs need to reduce students’ economic animosity to a level that does not manifest their overall animosity. To address this issue, the US HEIs should highlight the US-China economic relationship as a benign partnership rather than a rivalry so as to mitigate students’ economic animosity. Additionally, students’ overall animosity toward the US can also be triggered by religious animosity. Although Chinese students for the most part are atheists, exposure to negative US news media coverage regarding islamophobia in many Middle Eastern countries may still spark Chinese students’ religious antipathy toward studying in the US. Therefore, the US HEIs should promote and uphold policies to embrace diversity, especially among individuals who practice different faiths, and highlighting the benefits of learning and living in a diverse environment. As such, for US HEIs, students’ religious animosity should be contained below the threshold lest it manifests their overall animosity. Moreover, a high degree of military animosity is necessary for overall animosity to manifest. This result is positive news for US HEIs, given the fact that US government is dubbed infamously as the “world’s police”. The US HEIs need to make sure Chinese students do not expose any negative news regarding the US military’s involvement in the Asia-Pacific region.

Lastly, the US HEIs need to continue their promotional efforts in recruiting Chinese students in the future. The US HEIs are in the midst of a very intense competition. Based on this study’s descriptive analysis, more than half of the students expressed their interest in furthering their studies in Hong Kong or the UK. This suggests that US institutions operate in a competitive international education market and therefore need to differentiate themselves more effectively from other popular destinations. In this regard, the US HEIs may benefit from communicating concrete institution-level advantages, such as academic reputation, professional development opportunities, and student support, which may be more persuasive and credible than attempts to address macro-level political perceptions directly [18].

### Limitations and future research

These findings are subject to several limitations. The first limitation lies on this study’s sampling to generalize to similar contexts, as only cross-border Chinese undergraduate students in Macau were surveyed in this study. As such, due to potential same-source bias, the generalizability of this study is limited to specific student groups, especially those who study in Macau, SAR. Moreover, with a substantial proportion majoring in hospitality and tourism management, the findings may also not be generalizable to students in all majors. Therefore, future studies should examine students from diverse socioeconomic backgrounds at different Chinese mainland institutions, as Chinese students from different socioeconomic backgrounds may exhibit different perceptions and patterns in studying overseas [18]. Secondly, to not obscure the core mechanism (i.e., proposed hypothesized paths) due to the parsimonious nature of the theoretical framework, this study did not assess the direct effects of the proposed sub-dimensions of animosity constructs on risk perception, country image, and students’ study choice intention. However, future studies can develop a different conceptual framework to assess these direct relationships on the proposed outcomes to derive new theoretical insights. Future studies can also incorporate the concept of ethnocentrism along with animosity, as people can perceive ethnocentrism and animosity concurrently [13, 37]. Furthermore, the practical implications should be interpreted with caution, as statistical patterns may not provide an equivalent basis for identifying exact actionable causal levers. Additionally, this study dismissed concerns about visa policy constraints, career opportunities, and students’ levels of English proficiency on students’ study choice intentions. As such, future studies need to account for these unmeasured confounders to offer a more holistic picture of factors that are related to students’ perceptions and behaviors over studying abroad. Lastly, this study is based on quantitative methods with cross-sectional data, which may not provide a comprehensive analysis of students’ animosity and study abroad intention toward the US. Thus, future research can employ a longitudinal method to further explore the proposed relationships. Furthermore, experimental research with control variables or qualitative research methods, such as in-depth interviews, can be employed in future studies to provide a rich understanding of students’ perceptions.

## Conclusion

This research examined Chinese students’ psychological perceptions, specifically whether their sense of animosity could have any negative association with their decision-making in pursuing postgraduate education in the US. This research revealed that students’ feelings of overall animosity were largely attributed to the various levels of degree in political, historical, economic, social, religious, and military aspects of animosity. Students’ overall animosity is associated with their risk perception and their evaluation of the US, and consequently their decision-making about studying in the US. This research offered potential actionable implications for US HEIs to better understand students’ psychological thinking and mobility, helping them regain confidence to select the US as the host country for study.

## Supplementary Information

Supplementary Material 1.

## Data Availability

The datasets used and analyzed during the current study are available upon reasonable request.
