# Supplementary material for: Examining the psychological effects of animosity on university students’ study choice intention
Source: BMC Psychol. 2026 Apr 16;14:796. doi: 10.1186/s40359-026-04552-z (PMC13217940; doi:10.1186/s40359-026-04552-z)
Supplement: Supplementary file 1 — Supplementary Material 1. [file 40359_2026_4552_MOESM1_ESM.docx]

**Appendix 1. Measurement Items**

**Animosity** (Campo and Alvarez, 2019; Sanchez et al., 2018)

| ***Economic Animosity*** |
| --- |
| US is out to exploit the economy of my country and other counties |
| US is taking advantage of my country and other countries |
| Firms from US are doing business unfairly with China |
| ***Social Animosity*** |
| I dislike the mentality of the people of the US |
| I feel that people in the US are hostile towards my country |
| I dislike that people from the US Criticize my country’s policies. |
| ***Political Animosity*** |
| I dislike the policies of the government from the US |
| I dislike the political system in the US |
| I dislike the US because it does not respect human rights |
| ***Religious Animosity*** |
| US does not respect other religions |
| I dislike the religious system in the US |
| ***Historical Animosity*** |
| I dislike the US because of past historical events |
| I dislike the US because of its historic oppressing other countries |
| ***Military Animosity*** |
| I believe US poses a huge military threat |
| I dislike the US involvement in wars and conflicts |
| I dislike the military operations in the US |
| ***Overall Animosity*** |
| In general, I dislike the US |
| In general, I feel annoyed by the US |

**Country Image of the US** (De Nisco et al., 2016).

| US has high technology level |
| --- |
| US has advanced education level |
| US has high wealth |
| US has high quality of life |

**Risk Perception (**Tavitiyaman & Qu, 2013)

| Given the current US-China relation, traveling in the US is…. |
| --- |
| Given the current US-China relation, studying in the US is…. |

**Study Choice Intention** (Guo, 2025; To et al., 2014; Zhuang et al., 2015)

| I am interested in studying in the US |
| --- |
| I would apply a master’s program in the US |
| I plan to spend at least one semester studying in the US |
